# Complications From Transcatheter Pulmonary Valve Replacement With Self-Expanding Prestent

**DOI:** 10.1016/j.jaccas.2024.102836

**Published:** 2025-01-08

**Authors:** R. Allen Ligon, Stephen J. Nageotte, Ahmed Kheiwa, Robert J. Gray, Olivier Ghez, Ziyad M. Hijazi, Doff B. McElhinney, Vasilis C. Babaliaros, Dennis W. Kim, Dana M. Boucek, Brent M. Gordon

**Affiliations:** a Department of Pediatrics, Emory University School of Medicine, Atlanta, Georgia, USA; b Children’s Healthcare of Atlanta Cardiology, Atlanta, Georgia, USA; c Department of Pediatrics, Loma Linda University Children’s Hospital, Loma Linda, California, USA; d Department of Cardiology, Adult Congenital Heart Disease Program, Loma Linda University Medical Center, Loma Linda, California, USA; e Department of Pediatric Cardiology University of Utah and Primary Children's Hospital, Salt Lake City, Utah, USA; f Department of Cardiovascular Diseases, Sidra Medicine, Doha, Qatar; g Departments of Cardiothoracic Surgery and Pediatrics, Stanford University School of Medicine, Palo Alto, California, USA; h Division of Cardiology, Emory Structural Heart and Valve Center, Emory University Hospital Midtown, Atlanta, Georgia, USA; i Department of Pediatrics, Rady Children’s Hospital/University of California-San Diego School of Medicine, San Diego, California, USA

**Keywords:** Alterra Adaptive Prestent, Sapien S3, intravascular ultrasound, Papyrus coronary stent, pulmonary artery perforation, pulmonic valve, valve replacement

## Abstract

Self-expanding valve platforms are providing new and versatile options for transcatheter pulmonary valve replacement in patients with a large native or patched right ventricular outflow tract. We describe 4 cases of acute reintervention required after successful implantation of an Alterra Adaptive Prestent followed by SAPIEN S3.

## Visual Summary

**Figure:**
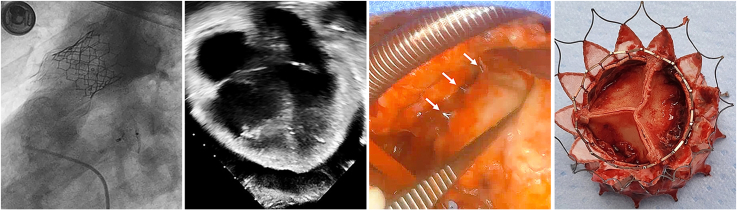

Transcatheter pulmonary valve replacement (TPVR) via a self-expanding platform represents a growing trend nationally.^1^, ^2^, ^3^, ^4^ Although procedural and short-term outcomes appear promising overall, complications other than ventricular arrhythmias are not well described.^5^ We report 4 patients who underwent successful TPVR using the Alterra Adaptive Prestent (AAP) and SAPIEN 3 (S3) valve (Edwards Lifesciences, Inc.) and who underwent subsequent intervention because of concerns for extravascular perforation of the main pulmonary artery (MPA) by tines of the AAP.

**Take-Home Messages**

• The Alterra Adaptive Prestent design and mechanism of fixation poses a risk of vascular perforation with potential clinical consequences; some patients may be at unique risk for clinically important pulmonary artery perforation.
• Advanced imaging is essential in the pre- and post-implantation management of patients with congenital heart disease undergoing TPVR.

## Patient 1

Patient 1 is a 23-year-old man who was born with valvar pulmonary stenosis (PS) and underwent pulmonary balloon valvuloplasty as a neonate. He developed free pulmonary regurgitation (PR) and severe right ventricular dilation and met screening criteria for TPVR with the AAP and S3.

**Figure 1:**
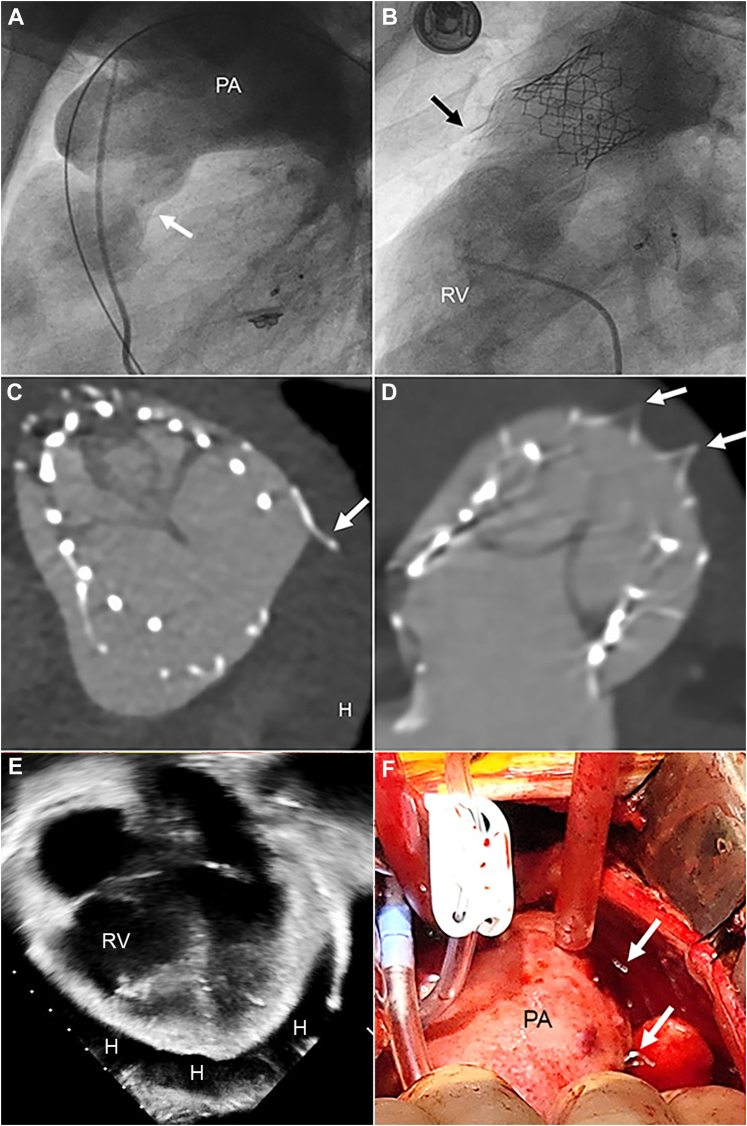
Patient 1 (A) This pulmonary arterial (PA) angiogram in the lateral projection demonstrates severe pulmonary regurgitation and the intended landing zone for the Alterra Adaptive Prestent in patient 1 (the arrow indicates the annulus). (B) This post-implant angiogram in the right ventricle (RV) demonstrates the Alterra Prestent and SAPIEN 3 valve in the supra-annular position. The proximal tines of the Alterra Prestent appear to be extravascular (arrow). (C and D) These (C) axial and (D) coronal cardiac computed tomography images obtained the day after implant demonstrate perforation of the distal tines of the Alterra Adaptive Prestent through the anteroleftward wall of the main PA (arrows), along with a moderate hemopericardium (H). (E) This 4-chamber echocardiographic image the day after implant demonstrates a large hemopericardium (H). (F) This intraoperative photograph reveals that the distal tines of the Alterra Prestent have perforated the left side of the main PA (arrows).

At cardiac catheterization, the AAP was advanced over a Lunderquist wire in the left pulmonary artery and deployed in the right ventricular outflow tract, covering the pulmonary valve leaflets (Figure 1A). After release, the inflow portion of the AAP appeared to be constrained and was dilated with a 25-mm Tyshak balloon to allow the device to completely expand. A 29-mm S3 valve was then deployed within the AAP. Post-implant angiography and intracardiac echocardiography demonstrated no PR, good flow into the branch pulmonary arteries, trivial para-Alterra leak, and no pericardial effusion (Figure 1B).

The patient’s postoperative course was complicated by chest pain; further evaluation with echocardiography demonstrated a moderate pericardial effusion without signs of tamponade. Cardiac computed tomography (CT) demonstrated moderate hemopericardium with multiple tips of the distal AAP extending outside the lumen of the MPA (Figures 1C and 1D). Pericardiocentesis was performed and 350 mL of blood was removed (Figure 1E).

After shared decision making with the patient and the surgical team, he underwent emergent surgery. At the time of surgery, frank blood was noted in the pericardium and 2 tines from the distal AAP were visible outside the MPA anteriorly and leftward (Figure 1F). The AAP and S3 percutaneous valve construct was easily removed. A 27-mm Inspiris Resilia valve was implanted, and the perforations of the MPA were oversewn. The patient was discharged home 2 days later. At follow-up 18 months after surgery, he continued to do well with no PS or PR and significant improvement in exercise tolerance.

## Patient 2

**Figure 2:**
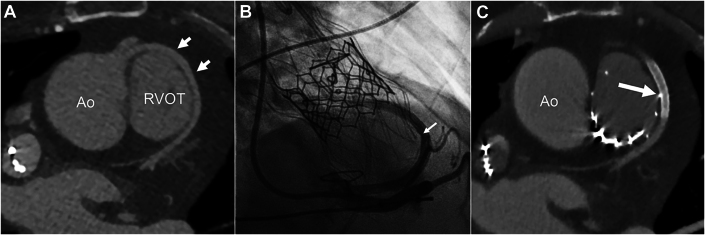
Patient 2 (A) This image from the preoperative computed tomography scan in patient 2 demonstrates the anomalous circumflex coronary artery originating from the right coronary artery and wrapping around the right ventricular outflow tract (RVOT) anteriorly (arrows). (B) This selective right coronary angiogram after placement of the Alterra Adaptive Prestent and SAPIEN 3 valve demonstrates focal compression of the anomalous circumflex artery by a proximal tine of the Prestent (small arrow). (C) This post-catheterization computed tomography image demonstrates a proximal tine of the Alterra Present projecting through the pulmonary artery and coming into contact with the stented anomalous circumflex (arrow). The portion of the coronary artery compressed by the tine now had covered stent material within it and was unobstructed. Ao = aorta.

A 49-year-old man with tetralogy of Fallot and anomalous origins of both the right and left coronary arteries from the anterior sinus underwent surgical repair as an infant. He subsequently developed right heart failure due to severe PR and right ventricular dilation with reduced function. CT demonstrated an extremely short left main coronary artery segment, and an anterior course of the left circumflex coronary artery (LCx), which wrapped around the right ventricular outflow tract before coursing posteriorly to travel in its usual location (Figure 2A).

The patient underwent cardiac catheterization for TPVR with the AAP and S3. Baseline coronary angiography and intravascular ultrasound (IVUS) imaging of the LCx were performed due to the CT findings. The AAP and valve were intentionally implanted high in the supra-annular position to avoid LCx compression and minimize interaction with the right ventricle. Pulmonary angiography after valve deployment demonstrated normal valve function with no residual PR. Repeat coronary angiography and IVUS suggested external compression of the proximal LCx by a tine from the AAP (Figure 2B, Video 1).

Because of concerns regarding risk for perforation, a 3.5 × 26-mm Papyrus covered coronary stent was deployed in the LCx during the same procedure, spanning the area of compression, under an emergency exception institutional review board protocol for this device. Repeat IVUS demonstrated a well-expanded stent throughout without any evidence of edge dissection. Post-procedure echocardiography demonstrated no pericardial effusion. However, CT confirmed one of the proximal AAP tines projecting out of the MPA and into the extravascular space adjacent to the anomalous LCx that was unobstructed and lined by the stent (Figure 2C).

At clinic follow-up 1 year later, the patient was asymptomatic and had normal pulmonary valve function. Right ventricular systolic function remains mildly reduced but his left ventricular function remains normal with no appreciable regional wall motion abnormalities. Scheduled 1-year CT coronary evaluation has yet to be performed.

## Patient 3

A 12-year-old boy with valvar PS underwent balloon valvuloplasty as an infant. He developed moderate to severe PR and right heart dilatation and was referred TPVR with the AAP and S3.

**Figure 3:**
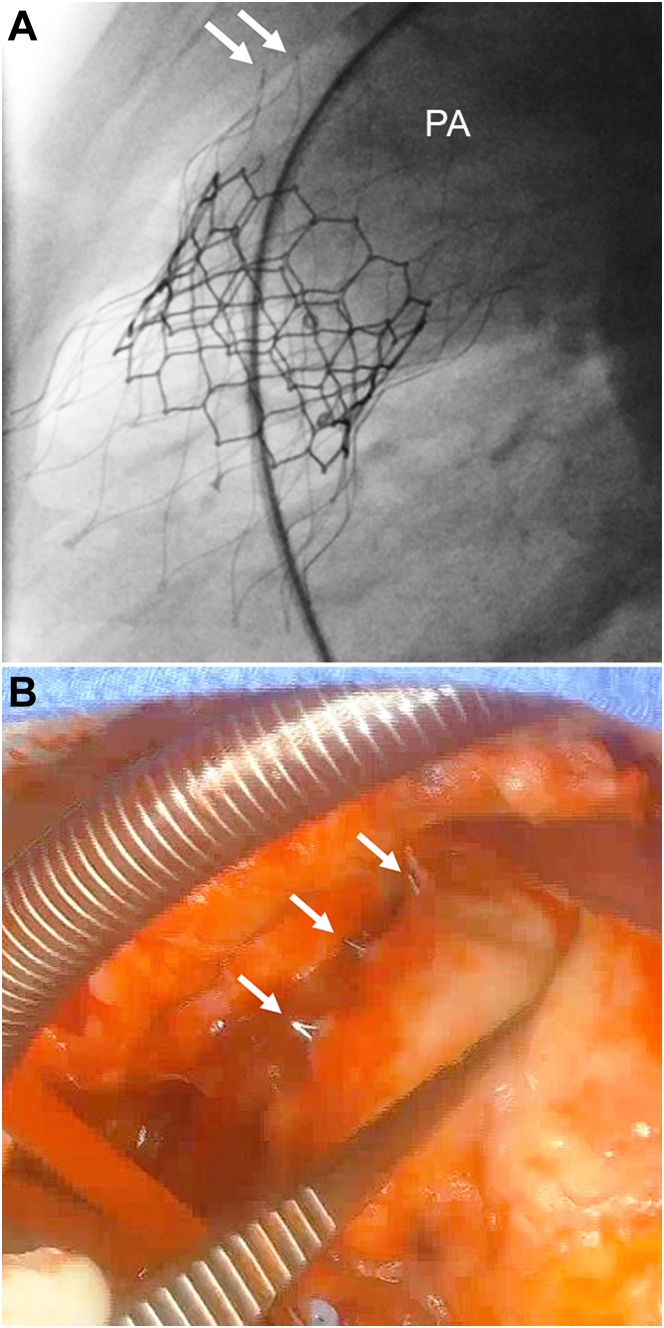
Patient 3 (A) This pulmonary artery (PA) angiogram obtained after valve implant shows 2 distal tines of the Alterra Adaptive Prestent tines appear to be protruding through the main PA (arrows). (B) This intraoperative photograph demonstrates extravascular protrusion of 3 distal tines (arrows) of the Alterra Adaptive Prestent that have perforated through the anterior wall of the main PA.

At cardiac catheterization, the AAP was deployed per standard technique in the annular position over a Lunderquist wire in the left pulmonary artery, followed by 29-mm S3 implantation. Post-implant angiography showed appropriate device position with no paravalvar or valvar regurgitation (Figure 3A).

The patient was hemodynamically stable but developed chest pain during recovery that was improved by laying prone and was treated with analgesics. An echocardiogram the next morning showed a large pericardial effusion without evidence of tamponade. A pericardial drain was placed, and 275 mL of blood was removed. As the patient was stable, the decision was made to manage the hemopericardium conservatively. The following day, a small pericardial effusion was noted so the drain was re-wired and 120 mL of serosanguineous fluid was drained. The drainage became serous and the drain was removed 2 days later. There was no re-accumulation of fluid on follow-up echocardiography and the patient was discharged home.

At follow-up 1 week later, the chest pain had resolved, and an echocardiogram showed a well-functioning pulmonary valve with no pericardial effusion. However, 3 weeks later he developed recurrent chest pain and was found to have a pericardial effusion. A CT scan showed that at least 1 tine of the AAP had perforated through the MPA and there was a 5- to 6-mm layer of pericardial fluid consistent with blood. The patient remained stable, and the decision was made to treat with steroids for presumed post-pericardiotomy syndrome. Over the following 6 months, he was treated several times for recurrent pericardial effusions that were minimally responsive to steroids. After his third recurrence, he was taken to surgery for explant of the AAP and S3 with pulmonary valve replacement. Intraoperatively, it was observed that 3 distal tines of the AAP had eroded through the anterior aspect of the MPA (Figure 3B). The AAP and S3 were removed, the perforations were repaired, and a 23-mm aortic homograft valved conduit was implanted. He did well after surgery and is currently being followed as an outpatient.

## Patient 4

**Figure 4:**
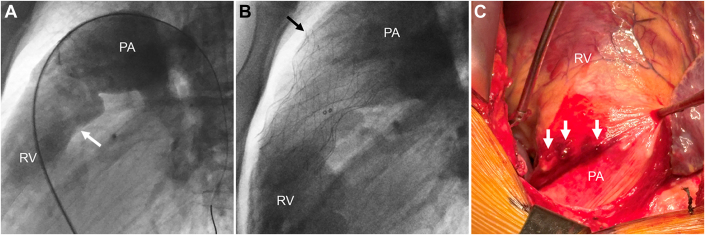
Patient 4 (A) This lateral-projection pulmonary artery (PA) angiogram in patient 4 demonstrates severe pulmonary regurgitation and the intended landing zone for the Alterra Adaptive Prestent (the arrow indicates the annulus). (B) This right ventriculogram following post-deployment of Alterra Adaptive Prestent demonstrates a well-seated device in the annular position. Note the interaction of the distal tines with the anterior portion of the main PA (arrow). (C) This intraoperative photograph of the right ventricular outflow tract demonstrates perforation of multiple tines of the Alterra Adaptive Prestent through the PA, with petechiae and hemorrhage of the PA (arrows).

A 25-year-old man with congenital valvar PS with no prior intervention(s) developed worsening PR with right ventricular dilation and met criteria for TPVR with AAP and S3. The fit analysis suggested that an annular implant would be feasible but with limited distal device apposition and potential risk of device embolization. Therefore, the plan was to perform staged TPVR and implant the S3 at a separate procedure. At cardiac catheterization, the AAP was deployed in an annular location over a Lunderquist wire in the left pulmonary artery and appeared to be stable (Figures 4A and 4B).

Shortly after the procedure, the patient developed significant chest pain, became tachycardic, then suffered a bradycardic arrest in the recovery room. Return of spontaneous circulation was achieved after chest compressions, intubation, and resuscitation medications. Echocardiography showed a large pericardial effusion that was drained at the bedside with removal of 350 mL of blood. The patient was taken immediately to the operating room for removal of the AAP, where 4 distal tines of the AAP were observed to have eroded anteriorly through the MPA (Figure 4C). The AAP was removed, the perforations were repaired, and a 29-mm Inspiris Resilia valve was implanted. The patient did well and was discharged home with a well-functioning pulmonary valve and good biventricular function.

## Discussion

In this case series, we report 4 patients who underwent intervention for clinically significant perforation of the MPA by tines of the prestent. All 4 were deemed by pre-procedural analyses to be appropriate candidates and underwent successful implantation by experienced implanters with placement of the device in the recommended location. The screening reports did not indicate excessive oversizing in any of these patients; in fact, the report for patient 4 actually suggested that the distal end of the device was borderline undersized. All patients underwent subsequent interventions because of concerns for perforation of the tines of the prestent through the MPA. Patients 1, 3, and 4 had native congenital PS and had not previously undergone cardiac surgery, so their mediastinum and heart were free of postoperative adhesions. Patient 2 also had evidence of the prestent tines protruding outside of the MPA, but he had undergone cardiac surgery and did not develop hemopericardium.

The critical role of cross-sectional and other advanced imaging modalities surrounding TPVR procedures cannot be overstated. Despite company review suggesting suitability for prestent and transcatheter valve in patient 2, careful pre-review of the CT identified the proximity of the LCx to the intended landing zone of the present, which prompted baseline selective coronary angiography and IVUS. Comparison of LCx imaging pre- and post-prestent implantation facilitated rapid identification of coronary artery compression with risk for perforation, and timely therapy with a covered stent. For all cases, CT imaging post-TPVR was able to identify protrusion of the prestent tines outside of the MPA.

The frequency of clinically relevant perforation of the MPA by the prestent is unknown, but a recent article reported protrusion of prestent tines through the pulmonary arterial wall detected on surveillance CT imaging in patients from 6 institutions.^6^ Those patients did not have clinical consequences. The authors reported 1 patient from each institution simply to illustrate this phenomenon but noted that there were more patients with this finding at all 6 centers. Thus, the incidence of silent perforation also remains to be determined, but it does not appear to be rare.

## Conclusions

Short-term results of TPVR with self-expanding valve platforms thus far have been outstanding and these devices promise to revolutionize the management of PR in patients with a large native or patched right ventricular outflow tract. The unique design of the AAP, with tines that protrude from the distal and proximal ends of the device to help secure it, may predispose this system to interact in unpredictable ways with the pulmonary artery or subvalvar right ventricular outflow tract. The risk of perforation and hemopericardium or other potential consequences should be considered, particularly in patients who have not undergone prior cardiac surgery. A high index of suspicion and judicious usage of adjunctive imaging is recommended for these patients. Larger studies with longer-term follow-up will be critical to evaluate the incidence and clinical significance of device erosion.

**Visual Summary:**
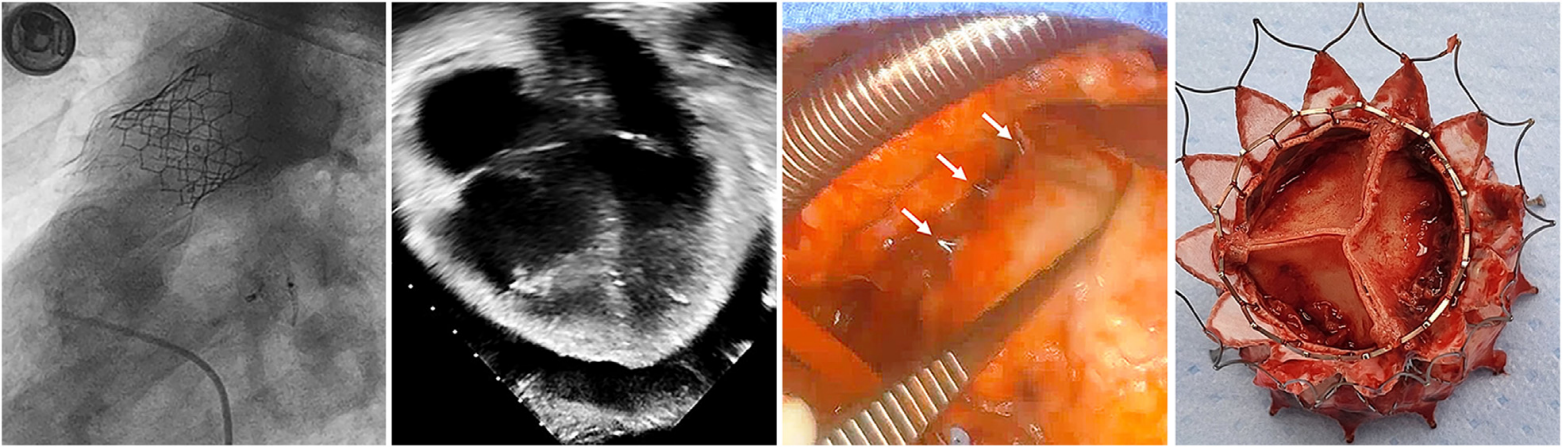
Self-Expanding Prestent Complications

## Funding Support and Author Disclosures

Dr McElhinney is a proctor and consultant for Edwards Lifesciences and Medtronic. Dr Babaliaros has received institutional research support and consulting fees from Edwards Lifesciences. Dr Kim has received consulting fees from Edwards Lifesciences. All other authors have reported that they have no relationships relevant to the contents of this paper to disclose.

## Appendix

Video 1 IVUS of left circumflex artery in Patient 2 IVUS imaging of the left circumflex artery after Alterra Adaptive Prestent and SAPIEN 3 valve placement. Note the region of the Alterra Prestent tine externally compressing the left circumflex artery (right side of screen, 3 to 5 o’clock position) throughout the cardiac cycle.
